# Interobserver and Intertest Agreement in Telemedicine Glaucoma Screening with Optic Disk Photos and Optical Coherence Tomography

**DOI:** 10.3390/jcm10153337

**Published:** 2021-07-28

**Authors:** Alfonso Anton, Karen Nolivos, Marta Pazos, Gianluca Fatti, Alejandra Herranz, Miriam Eleonora Ayala-Fuentes, Elena Martínez-Prats, Oscar Peral, Zaida Vega-Lopez, Antoni Monleon-Getino, Antonio Morilla-Grasa, Merce Comas, Xavier Castells

**Affiliations:** 1 Ophthalmology Department, Esperanza Hospital-Parc de Salut Mar, 08003 Barcelona, Spain; alejandra.herranz.cabarcos@gmail.com (A.H.); zaidaoptica@gmail.com (Z.V.-L.); 2 International University of Catalonia (UIC), 08017 Barcelona, Spain; 3 Institut Català de la Retina (ICR), 08017 Barcelona, Spain; frutalito@hotmail.com (M.E.A.-F.); amorilla@ioba.med.uva.es (A.M.-G.); 4 Department of Epidemiology and Evaluation, Hospital del Mar Institute for Medical Research, 08003 Barcelona, Spain; karenolivos@yahoo.es (K.N.); 93887@parcdesalutmar.cat (M.C.); XCastells@parcdesalutmar.cat (X.C.); 5 Department of Genetics, Microbiology, and Statistics, University of Barcelona, 08193 Barcelona, Spain; 6 Institut Clínic d’Oftalmologia (ICOF), Hospital Clínic de Barcelona, and Institut d’Investigacions Mèdiques August Pi iSunyer (IDIBAPS), 08036 Barcelona, Spain; martapazoslopez@gmail.com; 7 Hospital Vall d’Hebron, 08035 Barcelona, Spain; gianlucafatti@hotmail.com; 8 Barceloneta Primary Care Center, 08005 Barcelona, Spain; emartinez@perevirgili.cat; 9 Villa Olimpica Primary Care Center, 08005 Barcelona, Spain; operal@capvilaolimpica.net; 10 Section of Statistics (Department of Genetics, Microbiology, and Statistics), University of Barcelona, 08193 Barcelona, Spain; amonleong@ub.edu; 11 BIOST3 GRBIO (Research Group in Biostatistics and Bioinformatics), 08034 Barcelona, Spain; 12 Health Services Research on Chronic Patients Network (REDISSEC), Instituto de Salud Carlos III, 28029 Madrid, Spain

**Keywords:** screening, agreement, glaucoma, optical coherence tomography, retinographs, telemedicine

## Abstract

*Purpose*: To evaluate interobserver and intertest agreement between optical coherence tomography (OCT) and retinography in the detection of glaucoma through a telemedicine program. *Methods*: A stratified sample of 4113 individuals was randomly selected, and those who accepted underwent examination including visual acuity, intraocular pressure (IOP), non-mydriatic retinography, and imaging using a portable OCT device. Participants’ data and images were uploaded and assessed by 16 ophthalmologists on a deferred basis. Two independent evaluations were performed for all participants. Agreement between methods was assessed using the kappa coefficient and the prevalence-adjusted bias-adjusted kappa (PABAK). We analyzed potential factors possibly influencing the level of agreement. *Results*: The final sample comprised 1006 participants. Of all suspected glaucoma cases (*n* = 201), 20.4% were identified in retinographs only, 11.9% in OCT images only, 46.3% in both, and 21.4% were diagnosed based on other data. Overall interobserver agreement outcomes were moderate to good with a kappa coefficient of 0.37 and a PABAK index of 0.58. Higher values were obtained by experienced evaluators (kappa = 0.61; PABAK = 0.82). Kappa and PABAK values between OCT and photographs were 0.52 and 0.82 for the first evaluation. *Conclusion*: In a telemedicine screening setting, interobserver agreement on diagnosis was moderate but improved with greater evaluator expertise.

## 1. Introduction

The World Health Organization (WHO) reports that glaucoma is the second leading cause of blindness globally, after cataracts [1]. It is estimated that this disease affects 60 million people worldwide and it is expected to affect more than 80 million people in 2020 [2]. The prevalence of glaucoma in Spain is 2.1% in individuals older than 40 years of age [3]. In addition, the prevalence of glaucoma increases with age from 2.2% (50–59 years), 2.4% (60–69 years) to 3.7% in people older than 70 years. Moreover, because glaucoma is usually asymptomatic until advanced stages, the rate of undetected cases is very high, with reports of 67% in the UK [4] and 71% in Spain [3].

Glaucoma meets some of the criteria recommended by the WHO for consideration of useful and cost-effective screening programs. A previous study predicted that screening could be cost-effective in a 50-year-old cohort at a prevalence of 4% with a screening interval of at least two years [4].The question of which tests should be used to screen for glaucoma has no definite, scientifically proven, or even consensus-based answer [5]. Intraocular pressure (IOP) is usually included in screening protocols in combination with other tests to identify the most important risk factor, even though it has little value as a diagnostic test for glaucoma. Short functional tests of various kinds have proven useful for glaucoma screening, but they all have substantial disadvantages, including a relatively long testing time (one to several minutes) and the learning effect. These are particularly important for screening programs targeting a perimetrically-inexperienced population. All functional tests require the active participation by the individual being tested and repeat examinations [6] to overcome the learning effect. Additionally, they frequently have high false positive classification rates [6,7,8]. For these reasons [5,6,7,8], the acquisition of optic nerve and/or retinal nerve fiber layer photographs or images has become an increasingly used option for glaucoma screening. Additionally, many studies have demonstrated the capabilities of OCT to identify glaucomatous damage from early stages of the disease before functional tests can detect but OCT should not substitute visual fields in glaucoma management because the information given by functional and structural tests is additive [9]. Moreover, the use of OCT images together with visual fields in glaucoma follow up increases the chances to detect progression earlier [10]. Recently, a meta-analysis demonstrated that automated imaging is an effective aid to glaucoma diagnosis, and some imaging tests have been found to be adequate for cost-effective triage classification in the population at risk [11].

Additionally, glaucoma is especially suited for telemedicine (teleglaucoma) since tonometry, functional and/or structural tests can be performed at satellite sites and sent to the appropriate professionals for assessment with the purpose of screening in the population at risk or for patient follow-up over time [12]. In a systematic review evaluating the effectiveness of teleglaucoma for glaucoma screening, telemedicine was found to be advantageous in detecting true positive cases of glaucoma but had a higher rate of false positives than in-person examination [13]. Nevertheless, telemedicine for glaucoma screening has a demonstrated ability to detect glaucoma cases that may have been missed during in-person examination or at an earlier stage [14].

Finally, an instrument or procedure is accurate and reproducible if its results are consistent when applied more than once to the same individuals and under the same circumstances. The current study also focused on assessing interobserver agreement of image assessment in a screening setting using a customized telemedicine tool. The agreement between diagnostic tests and between two separate remote evaluations was analyzed during the process of classifying glaucoma suspects (binary scale). We also analyzed some potential factors influencing such agreement, such as participant age and physician experience.

The objectives of the present study were, first, to assess and compare the glaucoma detection rate of OCT and fundus photographs in a screening setting through a telemedicine program and, second, to evaluate the intertest and interobserver agreement.

## 2. Methods

### 2.1. Telemedicine Platform

The web-based telemedicine platform (DYSEO) was used and customized for this study. DYSEO is a cloud-based, store-and-forward tele-screening tool. It covers all the steps typically included in a screening program: patient recruitment, uploading of tests, remote evaluation of the studies, generation of diagnostic reports, integration of the reports with the medical record of the hospital, and monitoring with subsequent examinations in the hospital or in future campaigns. The tool allows secure storage of the images, tests and reports involved in the campaign in a remote cloud environment. DYSEO was designed following the model/controller/view (MVC) design pattern and was implemented using a web-based PHP open software framework. The views of the tool were built using modern web standards (HTML5, JavaScript and CSS technologies) and can be accessed in any tablet/desktop web browser with proper size configurations. The database was based in the open-source relational database management system MySQL and was deployed in a Linux server. The tool only allows access to previously authorized users with a login and password. All data transmission was encrypted bidirectionally with SSL (secure sockets layer) and the tool enforces integrity, confidentiality, availability, and resilience measures to protect the personal data stored in the application.

The remote center where examination and data collection took place was located at a primary care center (PCC). The activities carried out in the screening process included the generation of the agenda, examinations by optometrists, data and image upload, random assignment of images to ophthalmologists, and remote image evaluation and grading. Finally, DYSEO automatically generated a report based on the signs identified and ratings performed by the evaluators. The report was automatically sent to patients’ charts at the PCC and was notified to participants by surface mail.

### 2.2. Sample

The screening program was carried out in a population living along Barcelona’s coastline and included in the catchment area covered by two PCC, namely Barceloneta and Vila Olimpica. The sample size was calculated, for an overall evaluation of the screening method, and for 5% significance, 80% power, and a 1/2 ratio of individuals classified as positive/negative at screening, to detect a statistically significant difference in the area under the receiver operating characteristic (ROC) curve of 0.08 above 0.84 (that is, an area under the curve of 0.92). We estimated that 222 patients were needed, 74 screened as positive and 148 screened as negative. The reference value of 0.84 was obtained from the literature and, given that published studies were performed in selected populations and ours in the general population, we chose the lowest published accuracy value [15,16].

In this population-based study, the reference population included 18,185 men and women aged 55 to 85 years. A previous study performed in a similar setting obtained a low participation rate (25.5%) and a high percentage of losses for several reasons (incomplete or erroneous census information, inability to contact participants, deaths, and refusal to participate) [14]. Therefore, we estimated that it was necessary to randomly select over 4000 patients from the census to obtain a sample of 1000 examinations, of which we expected 7.7% to be identified as positive based on a previous population-based prevalence study performed in Spain [14]. The number of expected positive cases at screening was 77 and would cover the 74 estimated as the minimum sample needed (see above).

### 2.3. Examinations at Primary Care Centers

All individuals agreeing to participate were scheduled for an examination at their PCC, signed an informed consent before entering the study and completed a health questionnaire. Measures of visual acuity (with and without pinhole) and IOP with an air-puff tonometer (Topcon CT80, Itabashi-ku, Tokyo, Japan) were obtained. The study used the mean of two pressure measurements. Images of the optic disk, nerve fiber layer, ganglion cell complex at the macula, and standard macula images were obtained with a portable SD-OCT (iVue, Optóvue, CA, USA). Additionally, a fundus photograph including the optic disk and the macula was obtained with a non-mydriatic retinograph (Topcon TRC, Itabashi-ku, Tokyo, Japan). Data and images were uploaded in DYSEO and a request for evaluation was created in the “to do” list of one of the evaluators selected at random.

### 2.4. Image Evaluation and Grading

The images acquired at the PCC were remotely evaluated by a group of 16 evaluators, of whom nine were experienced physicians (over 10 years of practice) and six were young ophthalmologists or residents, and the DYSEO platform randomly assigned the cases among them. All evaluators classified the quality of each image as non-evaluable, poor, fair, or good. They were also required to assess the status of each image as non-useful, pathological, suspicious or with no signs of abnormality. Additionally, evaluators had to look for, and click on if found, any of the following signs: neuroretinal rim thinning, RNFL defect (RNFLD), peripapillary atrophy (PPA), and disk hemorrhage; they also had to estimate the vertical cup/disk ratio (C/D). An OCT or a photograph were considered as non-evaluable if the image was not uploaded or if the optic disk was not visible. Photographs were assigned one of quality degrees at evaluator’s criteria. An OCT was classified as poor if artifacts were present or quality index was under 45, as fair if quality index was between 46 and 60 and was qualified as having good quality if the mentioned index was over 60. Criteria to classify an OCT as suspicious was the presence of one of the following: RNFLD in thickness map, or global RNFL classification outside 99% normal limits, or at least one 90° RNFL sector outside 99% normal limits, or at least two 90° sectors were outside 95% normal limits. DYSEO tagged the case as “glaucoma suspect” if any image was classified as suspicious or pathological, and/or any glaucomatous sign was marked by the evaluator, and/or there was C/D asymmetry of more than 0.3 between the two eyes and/or the IOP was higher than 21 mmHg. Glaucoma suspects were recommended to undergo an ophthalmic examination.

For the analysis of interobserver agreement, all images were independently assessed by two different ophthalmologists and at a minimum interval of 1 month apart. If the two evaluators disagreed, a consensus evaluation was performed by two glaucoma experts (AA, EA). For patient classification purposes, the information from both eyes and all data and images (OCT and photographs) was considered, so when at least one eye was suspicious for glaucoma, the patient was considered a glaucoma suspect.

### 2.5. Statistical Analysis

Data from DYSEO was exported to an Excel sheet and all statistical analyses were performed with the free software R version 3.4.2 developed by the R Foundation for Statistical Computing (Vienna, Austria).

Descriptive statistics were applied to the data. The quality of images and detection rate of OCT and the fundus photograph were compared using the chi-squared test. Univariate analysis was performed to assess risk factors for being a glaucoma suspect. Kappa and PABAK were used to assess intertest and interobserver agreement on a binary scale (presence or absence of suspicion of the disease). The confidence intervals for each index were also reported as measures of statistical uncertainty. For comparative purposes, we tested the hypothesis that independent kappa estimates were equal, and the chi-squared test was used as defined by Fleiss [17]. Finally, Bland-Altman (B&A) analysis was used to assess interobserver agreement with quantitative measurements.

The kappa coefficient is a chance-corrected statistic widely used for measuring the level of agreement between raters for discrete outcomes (binary and categorical). Kappa ranges from −1 (perfect disagreement) to 1 (perfect agreement). A kappa coefficient of zero indicates no agreement better than simply applying chance to classify the cases. Kappa values were interpreted following the Landis classification [18]. Since kappa index can be significantly influenced by disease prevalence and glaucoma has a relatively low prevalence, PABAK was also used to adjust kappa values to the prevalence of case finding. PABAK also ranges from −1 to 1 and assumes an average of the prevalence of each category of the two raters. B&A plotting is a widely used method to assess interobserver agreement on a nominal scale. In the scatter plot, the average of two paired measurements can be visualized in the *x*-axis and the differences between these measurements in the y axis. The plot includes the average difference and the limits of agreement. B&A recommend that 95% of the data points should lie within ±2 standard deviations of the mean difference, under the assumption of normal distribution of the differences.

### 2.6. Ethics

This research study followed the tenets of the Declaration of Helsinki, and the study protocol was approved by the ethics committee of Parc Salut Mar.

## 3. Results

### 3.1. Study Sample

**Figure 1:**
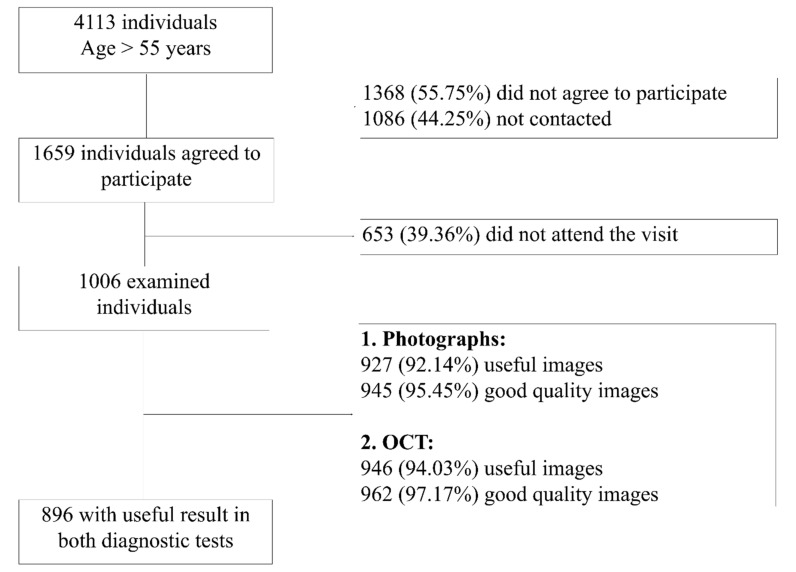
Distribution of sample through the screening process and the selection of sample subsets for agreement analysis.

Of theinitial sample of 4113 individuals, 1086 (26.4%) could not be reached and 1368 (33.3%) did not agree to participate in the screening program. In total, 1659 were scheduled for examination but only 1006 (24.5%) attended the visit and were examined at the PCC (Figure 1). Finally, 1006 participants (523 women and 483 men) with a mean age of 67 ± 7.8 years were included. Of them, 195 participants were older than 74 years. The characteristics of the sample are shown in Table 1. One hundred fifty participants (14.9%) were active smokers, 516 (51.3%) had systemic hypertension, 102 (10.1%) reported a family history of glaucoma, and 61 (5.1%) reported a prior diagnosis of glaucoma. Twenty-seven participants (2.7%) had IOPs greater than 21 mmHg, ranging from 22 to 27 mm Hg.

### 3.2. Image Quality

A non-significant tendency (*p* = 0.09) to a higher percentage of fair-good quality images and useful images was obtained with OCT (962 (97.2%) and 946 (94%), respectively) compared with fundus photographs (945 (95.5%) and 927 (92.1%), respectively). A total of 38 (34.5%) poor-quality images corresponded to participants over 74 years, while 31 (28.2%) were from the younger individuals (Figure 1). The frequency of poor-quality images increased significantly with increasing participant age (*p* < 0.0001).

### 3.3. Screening Results

Considering the consensus classification as the final screening result, the screening program identified 201 (19.9%) cases with suspicion of glaucoma and six could not be assessed due to the absence of useful images. On univariate analysis, the significant baseline risk factors for glaucoma suspicion were older age, higher IOP, low visual acuity, a personal history of ocular hypertension, and retinal surgery (Table 1). Of all participants identified as glaucoma suspects, 41 (20.4%) were identified in photographs only, 24 (11.9%) were identified in OCT images only, 93 cases (46.3%) in both type of images and 43 (21.4%) participants, despite being finally diagnosed with glaucoma, were not suspects in either of the two tests individually.

### 3.4. Interobserver Agreement Analysis

Of the sample, 436 (43%) participants were evaluated by two experienced ophthalmologists (experts) and 570 (57%) by an experienced and a younger ophthalmologist or resident (non-expert). The two classifications differed or disagreed in 238 (24%) cases. In those cases, the final classification was decided by consensus between two glaucoma experts (AA, MEA). For this agreement analysis, a subset sample was created with all persons with both good quality and useful images in OCT and photographs (*n* = 896). In total, 110 cases (10.9%) were excluded due to poor quality or not useful image of any type. Sixty-three patients were excluded from this analysis because of useful but poor-quality photographs, 31 because of useful but poor-quality OCT images, and 13 participants had no OCT images. The overall proportion of agreement in the final screening classification was 0.79. The kappa coefficient was 0.37 (CI: 0.29–0.44), but the PABAK index, corrected for prevalence, was 0.58 (CI: 0.52–0.64). The results of interobserver agreement in relation to evaluator experience and type of test are shown in Table 2. In general, agreement was approximately 0.6 or less. In terms of the overall classification, there was a tendency for greater agreement between two experienced evaluators (the kappa coefficient was 0.39 (CI: 0.28; 0.51) and PABAK index was 0.66 (CI: 0.56; 0.75)) than when one less experienced evaluator participated (with a kappa of 0.35 (0.26; 0.45) and PABAK index of 0.52 (CI: 0.43; 0.61)). The interobserver agreement in OCT evaluation showed a kappa coefficient of 0.51 (CI: 0.43; 0.60) and a PABAK index of 0.78 (CI: 0.72; 0.83), which tended to be greater than the kappa 0.37 (CI: 0.29–0.44) and PABAK index 0.58 (CI: 0.52; 0.64) obtained with photographs. The highest agreement value was obtained when OCT was assessed by two experienced ophthalmologists with a kappa coefficient of 0.61 (CI: 0.49; 0.73) and a PABAK of 0.82 (CI: 0.74; 0.89).

**Figure 2:**
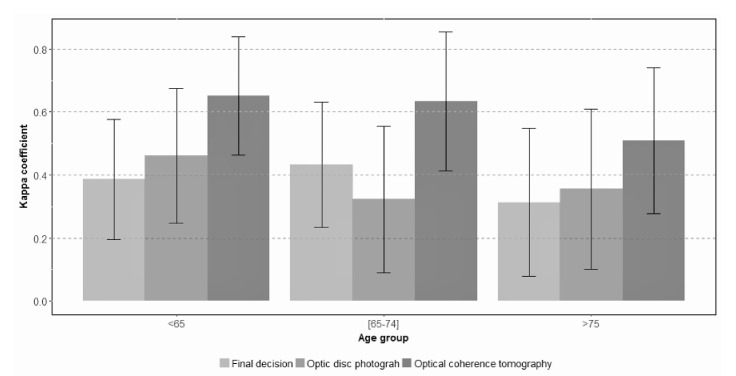
Interobserver agreement between two expert evaluators in each age group. The y axis shows kappa coefficient estimates and their respective confidence intervals. The x -axis shows the three age groups: under 65 years, 65 to 74 years, and over 74 years. Kappa values tend to be greater with younger participant age and on evaluation of OCT images vs. photographs.

**Figure 3:**
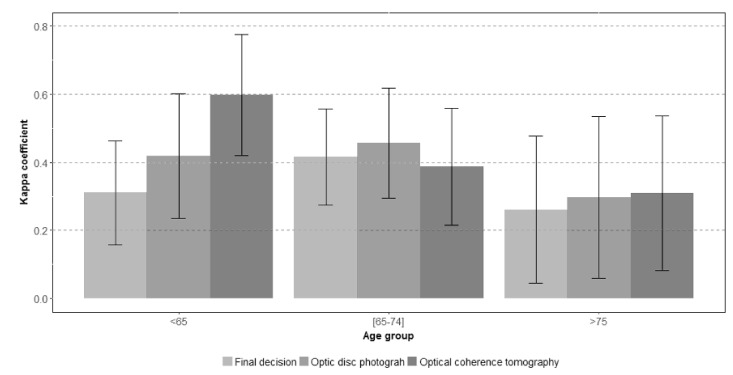
Interobserver agreement between expert and non-expert evaluators in each age group. The y axis depicts the kappa coefficient estimates and their respective confidence intervals. The *x*-axis shows the three age groups: under 65 years, 65 to 74 years, and over 74 years. Kappa values are highest on evaluation of OCT images from the youngest participants and decrease with increasing age. The lowest agreement values were found within the oldest group of participants for overall, OCT and photograph evaluation.

Figure 2 (expert vs. expert) and Figure 3 (expert vs. non-expert) show the degree of agreement according to evaluator expertise in each age group.Among the 394 participants younger than 65 years old, the overall proportion of agreement was 0.81 and the proportion of positive cases was 7%. The kappa coefficient obtained for the final screening decision was 0.34 (CI: 0.22; 0.46), while PABAK was 0.62 (CI: 0.57; 0.67). Forty two percent of participants in the youngest group (55 to 64 years) was assessed by two experienced ophthalmologists and, in them, the agreement obtained was slightly higher, with a kappa coefficient of 0.39 (CI: 0.19; 0.58) and a PABAK index of 0.70 (CI: 0.45; 0.95). The remaining 58% of participants, in the youngest group, were assessed by two ophthalmologists with different levels of expertise and the kappa coefficient was 0.31 (CI: 0.16; 0.46).If the interpretation of the two diagnostic tests was considered separately, photographs and OCT, the degree of agreement tended to be greater than the agreement in the overall screening classification. The highest kappa value reached was 0.65 (CI: 0.46; 0.84) in the assessment of OCT tests by two expert ophthalmologists (Figure 3).

In the 345 participants aged 65–74 years old, interobserver agreement showed a kappa coefficient of 0.43 (CI: 0.32; 0.55). Again, 43% of cases were assessed by two expert ophthalmologists. Nevertheless, independently of the level of expertise, the agreement estimated by kappa did not change across diagnostic tests (OCT vs. photos) and remained around 0.43. However, with the PABAK index, the agreement tended to be lower when a non-expert evaluator intervened in the process.

Finally, and as expected, the overall proportion of global classification agreement was lower, and the proportion of positive cases was higher in the group with the oldest participants (≥75 years). Analysis of the evaluations in the 157 oldest subjects revealed that the kappa coefficient was the lowest with a value of 0.28 (CI: 0.08; 0.45) and increased to 0.40 (CI: 0.28; 0.52) if the PABAK index was calculated. Of this group of participants, 72 (45.8%) were assessed by two experts and the agreement between them was 0.31 (CI: 0.08; 0.55), the lowest among the three age groups. Finally, the degree of agreement tended to be higher in the evaluation of OCT images with a kappa of 0.51 (CI: 0.19; 0.72) and PABAK index of 0.88 (CI: 0.73, 1.00). In accordance with all these findings, agreement decreased with increasing participant age in the evaluation of both tests, photographs (*p* < 0.0001), and OCT images (*p* < 0.0001). This last significant tendency was also observed even when the assessment was carried out by two expert evaluators (*p* < 0.0001).

### 3.5. Intertest Classification Agreement

During the first evaluation, the classification of photographs and OCTs agreed in 814 cases (90.8%), of which 7% were classified with glaucoma suspicion and 93% were classified as normal. For this first evaluation, the kappa coefficient was 0.52 (CI: 0.43; 0.61) and the PABAK was 0.82 (0.77; 0.87). During the second evaluation round, the evaluation of photographs and OCTs agreed in 778 (86.8%) cases, of which 11% were classified with glaucoma suspicion and the remaining cases (89%) were classified as normal; the kappa index was 0.51 (CI: 0.43; 0.59) and the PABAK index was 0.72 (CI: 0.66; 0.78).

## 4. Discussion

Telemedicine has now been used for decades in ophthalmology, especially in the detection of diabetic retinopathy. However, its use in glaucoma is far from widespread, despite its potential benefits for screening and follow-up. A meta-analysis evaluating the effectiveness of teleglaucoma for screening concluded that it could detect more cases of glaucoma than in-person examination [13]. The most frequent screening tests are optic nerve photographs, IOP, and visual fields. Undoubtedly, the use of imaging devices, specifically OCT, has progressively increased in the last two decades. However, there is no ideal single test for the purpose of glaucoma screening [5] and the combination of tests used depends on organizational resources, target goals, and populations.

The present study assessed tests and evaluator agreement during glaucoma screening in a population-based sample. A telemedicine program with OCT, fundus photographs and intraocular pressure was implemented without the use of functional tests. In this setting, the detection rate was 19%, which is lower than the 28% of suspicious retinographs found in the Philadelphia Telemedicine Glaucoma Detection and Follow-up study [19]. This is likely because their screening targeted an even higher risk population with greater chances of having glaucoma than the population aged over 55 years chosen for this study. We specifically evaluated the interobserver and intertest agreement and the influence of image quality, evaluator experience and population age on such agreement. Being a population-based study offers the chance to evaluate tests and evaluator abilities in the environment and setting where these activities are most likely to be performed. The authors decided to include persons with history of glaucoma or ocular hypertension for three reasons. First, the source of that information was the patient or the family and this may be inaccurate. Second, preserving them in the sample helps to maintain the population-based condition of the sample. Finally, not all persons who think to have glaucoma have been adequately tested and diagnosed. The only drawback of including cases with personal positive history is the potential bias of over-assessing the detection rate results, but this seems unlikely since family or personal history were not considered by image evaluators.

There are several statistical indices for the assessment of agreement. The kappa coefficient is the most popular index for measuring agreement between discrete outcomes, due to its simplicity, applicability, and intuitive explanation. Nevertheless, several limitations of this coefficient have been published in the literature [20,21], the most important being the significant influence of low disease prevalence on kappa values [22,23]. For this reason, in the present study we also calculated the prevalence-adjusted bias-adjusted kappa index (PABAK). Overall interobserver agreement outcomes were a kappa coefficient of 0.37 and a PABAK index of 0.58. As expected, because the prevalence of positive screening cases was relatively low, PABAK values were higher than kappa values for almost all parameters. On comparison of interobserver agreement among different age groups of participants, PABAK provided significant adjustments, with the exception of the older groups in which both kappa and PABAK values were similar, probably due to the increase in prevalence that occurs with age.

We also analyzed the various factors influencing agreement and found that older participant age and lower evaluator experience were significantly associated with worse kappa estimates. These findings emphasize two important issues. First, the single-reader approach of many screening programs should probably be reconsidered to optimize the classification results. Possibly, with non-expert evaluators, a double reading would be recommended, especially in older patients, who have a higher risk of evaluator disagreement. Second, evaluation is more difficult and less reproducible in elderly patients probably because they more often have lens opacities and high-quality images are harder to obtain.

As a further demonstration of the undoubtedly present interobserver variability, there were some mild agreement differences between the results obtained in the first and the second assessment rounds. This was expected both in general and in particular because the cases were randomly distributed among the evaluators. Regarding OCT and retinograph intertest agreement, kappa coefficient and PABAK estimates between OCT and photographs were 0.52 and 0.82 for the first evaluation and 0.51 and 0.72 after the second, respectively.

On comparison of the two tests involved in this screening program, OCT showed a greater degree of agreement between evaluators, while retinographs demonstrated a higher variability in the results and lower agreement. This was an unsurprising finding since OCT offers an objective classification and color-coded maps, while optic disc photograph assessment is probably a more demanding activity depending completely on the evaluator’s knowledge and experience. During our study, a guide showing evaluation criteria was available for evaluators, who were encouraged to follow it; nevertheless, interobserver agreement was only moderate. In our study, the highest agreement values were obtained when OCT was assessed by two experienced ophthalmologists (kappa = 0.61; PABAK = 0.82).

Previous studies have shown that including OCT in telemedicine equipment may improve classification [13] and the reproducibility of assessments. Our results support a greater agreement among evaluations when OCT is used, compared with photographs, which would probably favor a higher reproducibility of glaucoma screening classifications. Results on the cost-effectiveness of using OCT for glaucoma screening vary. One study found it not to be cost-effective when used as a triage test [11], while another study including OCT demonstrated that implementing teleglaucoma was cost-effectivein a rural population at risk of glaucoma [2]. Since portable and cheaper OCTs are currently available, new, and more complete cost-effectiveness studies on glaucoma screening with OCT are needed. OCT has nevertheless some limitations that explain, at least in part, why sensitivity and specificity to detect glaucomatous damage is not 100%. Firstly, results are dependent and can be significantly influenced by image quality and the presence of artifacts. Secondly, retinal anatomy is considerably heterogeneous. Thirdly, normative databases are limited in number, and have difficulty in including adequate representation of all heterogeneous forms and sizes of disks, ethnical groups, or retinal anatomies. It is very difficult to obtain good quality OCT image in high myopic eyes, and almost impossible to obtain a reliable automatic classification even using the recently developed minimum rim width parameter [24] since OCT databases do not include those cases and their RNFL (thinner and with more distance between humps) and optic disc anatomy (peripapilar atrophy, greater disk size…) are very different from that of emmetropic eyes.

The use of two or more different tests for glaucoma screening in the same population increases the chances to identify glaucomatous damage but also increases the chances for disagreement among the tests used. As explained in the introduction, there are objective reasons to use imaging devices added to tonometry and to exclude functional tests for screening purposes. The later are very useful for glaucoma diagnosis and follow up but quite unpractical in a screening setting due to test time and learning effect. Whatever tests are used, their characteristics will directly influence the sensitivity and specificity to detect cases, as well as the reproducibility of the results. In our study, the addition of OCT to fundus photographs seems to allow the detection of a significant number of cases that would not have been identified if only one test had been used. In this particular setting and sample the authors would recommend, and so was done during the study, to consider suspect any eye with signs of glaucoma in fundus photographs and/or in OCT images.

Nowadays the task of image evaluation is being progressively transferred to automatic, artificially intelligence (IA)-based, algorithms. Nevertheless, very few of them have being widely implemented and, to the best of our knowledge, no IA algorithm for identifying glaucomatous damage has been approved for clinical use. For this reason, characterizing the precision and limitations of subjective evaluation of photographs and OCT images is of undoubtful clinical interest. The same images obtained in this study were evaluated by our own convolutional neural network, which was able to classify cases with an area under the ROC curve of 0.85 (Figure 9 from Gomez-Valverde et al., 2019) [25].

This study has some limitations, which did not preclude it from achieving its objectives. First, the evaluators participating in the study were assigned cases randomly and in a competitive manner, so the distribution of cases per evaluator was not necessarily homogeneous, with half of all cases being assigned to only five of 16 evaluators. The authors believe that the random distribution of cases to evaluators adds robustness to the method. Second, the percentage of patients who could not be reached by telephone and the patient drop-out rate were high. Although not surprising for a population-based study, this could have affected the characteristics of the randomly selected sample. However, when we compared the age and gender distribution of the final sample obtained to that of the original population, we found no statistically significant differences. Third, the sample calculation was performed to identify small classification differences, because this is part of a larger study evaluating the accuracy and cost of different screening methods for glaucoma. Nevertheless, over 1000 randomly selected cases appeared to be a solid source of data for the purposes of this study.

In summary, the screening program identified glaucoma suspects in 19.9% of the cases examined. Interobserver agreement was moderate (0.41–0.60) to substantial (0.61–0.80) in most cases but only fair in some specific subgroups. Agreement between photographs and OCT images was moderate but seemed to provide additional information. Participant age and evaluator expertise can significantly influence screening results. Even though the question of which tests should be used to screen for glaucoma has no definite, scientifically proven, or even consensus-based answer [5], a study like ours shows some evidence about the usefulness and limitations of screening for glaucoma using imaging devices.

## Figures and Tables

**Table 1:** Sample characteristics.

Categorical Variable	Categories	Total Group (*n* = 1006)	Suspects (*n* = 201) (19.9%)	Non-Suspects (*n* = 799) (79.4%)	*p*-Value
Gender	Female	523 (51.9)	94 (46.8)	426 (52.9)	0.2503
	Male	483 (48.1)	107 (53.2)	373 (47.1)	
Age	<65	425 (42.2)	64 (31.8)	360 (45.1)	<0.001
	65–74	386 (38.4)	81 (40.3)	305 (38.2)	
	>74	195 (19.4)	56 (27.9)	134 (16.7)	
Visual acuity	Low (<0.2)	63 (6. 3)	31 (15.4)	31 (15.4)	<0.001
	Medium ([0.2–0.5])	166 (16.5)	41 (20.4)	41 (20.4)	
	High (>0.5)	777 (77.2)	129 (64.2)	129 (64.2)	
Intraocular pressure	(0–21)	979 (97.3)	178 (88.6)	795 (99.5)	< 0.001
	>21	27 (2.7)	23 (11.4)	4 (0.5)	
Smoker type	Active	150 (14.9)	27 (13.4)	122 (15.3)	0.5077
	Former	338 (33.6)	74 (36.8)	261 (32.7)	
	Never	518 (51.5)	100 (49.8)	416 (52.0)	
Hypertension	yes	516 (51.3)	108 (53.7)	406 (50.8)	0.4594
Hypertension control	Inadequate control	21 (2.1)	3 (1.5)	18 (2.3)	
	Adequate control	482 (47.9)	98 (48.8)	382 (47.8)	0.7893
	Not under follow up	503 (50.0)	100 (49.7)	399 (49.9)	
Stroke	yes	46 (4.6)	11 (5.5)	35 (4.4)	0.5088
Myocardial infarction	yes	93 (9.24)	24 (11.9)	69 (8.6)	0.1493
Hyperlipidemia	yes	421 (41.8)	79 (39.3)	340 (42.6)	0.4039
Family history of glaucoma	yes	102 (10.1)	13 (6.5)	89 (11.1)	0.0504
Personal record of glaucoma	yes	61 (5.1)	24 (11.9)	34 (4.3)	<0.001
Personal history of ocular hypertension	yes	29 (2.9)	10 (4.9)	19 (2.4)	0.0498
Glaucoma laser therapy	yes	11 (1.1)	2 (0.9)	9 (1.1)	0.8732
Retina laser therapy	yes	36 (3.6)	7 (3.5)	29 (3.6)	0.5432
Retina surgery	yes	19 (1.9)	12 (5.9)	7 (0.9)	0.0022
Refractive surgery	yes	21 (2.1)	3 (1.5)	18 (2.3)	0.5654

The screening program identified 201 (19.9%) cases with suspicion of glaucoma and 6 could not be assessed due to absence of useful images. According to a univariate analysis, the significant baseline risk factors for glaucoma suspicion were older age, higher IOP, low acuity visual, a personal history of ocular hypertension or glaucoma, and previous retinal surgery.

**Table 2:** Agreement in test evaluation and medical findings (overall and by experience).

Overall Level	Tests			Medical Findings			
	Final Decision	Photos	OCT	Rim Thinning	RNFL Defect	Disc Hemorrhage	Parapapillary Atrophy
***p_o_***	0.79	0.86	0.89	0.90	0.93	0.99	0.87
***κ***	0.37	0.41	0.51	0.30	0.12	0.61	0.18
***κ_pb_***	0.58	0.72	0.78	0.80	0.86	0.98	0.74
**Expert-Expert**							
***p_o_***	0.83	0.88	0.91	0.93	0.95	0.99	0.87
***κ***	0.39	0.39	0.61	0.46	0.20	0.66	0.13
***κ_pb_***	0.66	0.76	0.82	0.86	0.90	0.98	0.74
**Expert–Non-expert**							
***p_o_***	0.76	0.85	0.87	0.88	0.93	0.99	0.87
***κ***	0.35	0.42	0.45	0.18	0.07	0.57	0.21
***κ_pb_***	0.52	0.70	0.74	0.76	0.86	0.98	0.74

RNFL: retinal nerve fiber layer; is the overall proportion of agreement; is the kappa coefficient; is the prevalence-adjusted bias-adjusted kappa (PABAK). Legend Table 2. There was a tendency for greater agreement between two experienced evaluators than when one less experienced evaluator participated. The interobserver agreement in OCT evaluation tended to be greater than that obtained with photographs. The highest agreement was obtained when OCT was assessed by two experienced ophthalmologists.

## Data Availability

Dataare safely kept and anonymized but havenot been made available.

## References

[1] Resnikoff S., Pascolini D., Etya’ale D., Kocur I., Pararajasegaram R., Pokharel G.P., Mariotti S.P. (2004). Global Data on Visual Impairment in theYear 2002. Bull. World Health Organ..

[2] Thomas S., Hodge W., Malvankar-Mehta M. (2015). TheCost-Effectiveness Analysis of Teleglaucoma Screening Device. PLoS ONE.

[3] Antón A., Andrada M.T., Mujica V., Calle M.A., Portela J., Mayo A. (2004). PrevalenceofPrimary Open-Angle Glaucoma in a Spanish Population: The Segovia Study. J. Glaucoma.

[4] Burr J., Mowatt G., Hernández R., Siddiqui M., Cook J., Lourenco T., Ramsay C., Vale L., Fraser C., Azuara-Blanco A. (2007). The Clinical Effectiveness and Cost-Effectivenessof Screening for Open Angle Glaucoma: A SystematicReview and Economic Evaluation. HealthTechnol. Assess. (Rockv).

[5] Mowatt G., Burr J.M., Cook J.A., Siddiqui M.A.R., Ramsay C., Fraser C., Azuara-Blanco A., Deeks J.J. (2008). Screening Testsfor Detecting Open-Angle Glaucoma: Systematic Review and Meta-Analysis. Investig. Opthalmology Vis. Sci..

[6] KamdeuFansi A.A., Li G., Harasymowycz P.J. (2011). TheValidityof Screening For Open-Angle Glaucoma in High-Risk Populations With Single-Test Screening Mode Frequency Doubling Technology Perimetry (FDT). J. Glaucoma.

[7] Wessels I.F., Randhawa R.S. (1996). Improving the Sensitivity of the OKP Visual Field Screening Test with a Blue Stimuluson a DarkBackground. Eye.

[8] De Tarso Ponte Pierre-Filho P., Schimiti R.B., De Vasconcellos J.P.C., Costa V.P. (2006). Sensitivity and Specificity of Frequency-Doubling Technology, Tendency-OrientedPerimetry, SITA Standard and SITA FastPerimetry in Perimetrically Inexperienced Individuals. Acta Ophthalmol. Scand..

[9] Gray J., McMeekin P., Hernández R., Cook J., Burr J.M., Ramsay C., Garway-Heath D., Gray J., Mc Meekin P., Hernández R. (2016). Can Automated Imaging for Optic Disc and Retinal Nerve Fiber Layer Analysis Aid Glaucoma Detection?. Ophthalmology.

[10] Garway-Heath D.F., Zhu H., Cheng Q., Morgan K., Frost C., Crabb D.P., Ho T.-A., Agiomyrgiannakis Y. (2018). Combining Optical Coherence Tomography with Visual Field Data to Rapidly Detect Disease Progression in Glaucoma: A Diagnostic Accuracy Study. HealthTechnol. Assess. (Rockv).

[11] Azuara-Blanco A., Banister K., Boachie C., Mcmeekin P., Gray J., Burr J., Bourne R., Garway-Heath D., Batterbury M., Hernández R. (2016). Automated Imaging Technologies for the Diagnosis of Glaucoma: A Comparative Diagnostic Study for the Evaluation of the Diagnostic Accuracy, Performance as Triage Tests and Cost-Effectiveness (GATE Study). Health Technol. Assess. (Rockv).

[12] Keenan J., Shahid H., Bourne R.R., White A.J., Martin K.R. (2015). Cambridge Community Optometry Glaucoma Scheme. Clin. Experiment. Ophthalmol..

[13] Thomas S.M., Jeyaraman M., Hodge W.G., Hutnik C., Costella J., Malvankar-Mehta M.S. (2014). The Effectiveness of Teleglaucoma versus In-Patient Examination for Glaucoma Screening: A Systematic Review and Meta-Analysis. PLoS ONE.

[14] Anton A., Fallon M., Cots F., Sebastian M.A., Morilla-Grasa A., Mojal S., Castells X. (2017). Cost and Detection Rate of Glaucoma Screening with Imaging Devices in a PrimaryCare Center. Clin. Ophthalmol..

[15] Fallon M., Valero O., Pazos M., Antón A. (2017). Diagnostic Accuracy of Imaging Devices in Glaucoma: A Meta-Analysis. Surv. Ophthalmol..

[16] Michelessi M., Lucenteforte E., Oddone F., Brazzelli M., Parravano M., Franchi S., Ng S.M., Virgili G. (2015). Optic Nerve Head and Fibre Layer Imaging for Diagnosing Glaucoma. Cochrane Database Syst. Rev..

[17] Fleiss J.L. (1981). Statistical Methods for Rates and Proportions, 2d ed..

[18] Landis J.R., Koch G.G. (1977). The Measurement of Observer Agreement for Categorical Data. Biometrics.

[19] Hark L.A., Katz L.J., Myers J.S., Waisbourd M., Johnson D., Pizzi L.T., Leiby B.E., Fudemberg S.J., Mantravadi A.V., Henderer J.D. (2017). Philadelphia Telemedicine Glaucoma Detection and Follow-up Study: Methods and Screening Results. Am. J. Ophthalmol..

[20] Banerjee M., Capozzoli M., McSweeney L., Sinha D. (1999). Beyond Kappa: A Review of Interrater Agreement Measures. Can. J. Stat..

[21] Shoukri M.M. (2004). Measures of Interobserver Agreement.

[22] Feinstein A.R., Cicchetti D. (1990). V High Agreementbut Low Kappa: I. the Problems of Two Paradoxes. J. Clin. Epidemiol..

[23] Byrt T., Bishop J., Carlin J.B. (1993). Bias, Prevalence and Kappa. J. Clin. Epidemiol..

[24] Chauhan B.C., O’Leary N., Almobarak F.A., Reis A.S.C., Yang H., Sharpe G.P., Hutchison D.M., Nicolela M.T., Burgoyne C.F. (2013). Enhanced Detection of Open-Angle Glaucoma withan Anatomically Accurate Optical Coherence Tomography-Derived Neuroretinal Rim Parameter. Ophthalmology.

[25] Gómez-Valverde J.J., Antón A., Fatti G., Liefers B., Herranz A., Santos A., Sánchez C.I., Ledesma-Carbayo M.J. (2019). Automatic Glaucoma Classification Using Color Fundus Images Basedon Convolutional Neural Networks and Transfer Learning. Biomed. Opt. Express.

